# Facile synthesis of PEG-glycerol coated bimetallic FePt nanoparticle as highly efficient electrocatalyst for methanol oxidation

**DOI:** 10.1038/s41598-023-38358-5

**Published:** 2023-08-15

**Authors:** Sarmistha Baruah, Barkha Rani, Niroj Kumar Sahu

**Affiliations:** 1 Centre for Nanotechnology Research, Vellore Institute of Technology, Vellore, 632014 India; 2 School of Electronics Engineering, Vellore Institute of Technology, Vellore, 632014 India

**Keywords:** Nanoscale materials, Materials for energy and catalysis, Nanoscale materials

## Abstract

Direct methanol fuel cell (DMFC) has shown excellent growth as an alternative candidate for energy sources to substitute fossil fuels. However, developing cost-effective and highly durable catalysts with a facile synthesis method is still challenging. In this prospect, a facile strategy is used for the preparation of hydrophilic Fe-Pt nanoparticle catalyst via a polyethylene glycol-glycerol route to utilize the advantages of nanostructured surfaces. The synthesized electrocatalysts are characterized by XRD, XPS, TEM, EDS and FTIR to confirm their structure, morphology, composition, and surface functionalization. The performance of the catalysts towards methanol oxidation reaction (MOR) was investigated by cyclic voltammetry and chronoamperometry in both acidic and alkaline media. The Fe-Pt bimetallic catalyst exhibits better current density of 36.36 mA cm^−2^ in acidic medium than in alkali medium (12.52 mA cm^−2^). However, the high I_f_/I_b_ ratio of 1.9 in alkali medium signifies better surface cleaning/regenerating capability of catalyst. Moreover, the catalyst possessed superior cyclic stability of ~ 80% in the alkaline electrolyte which is 1.6 times higher than in the acidic one. The better stability and poison tolerance capacity of catalyst in alkaline media is attributed to the OH^−^ ions provided by the electrolyte which interact with the metal species to form M-(OH)_x_ and reversibly release OH^−^ and regenerate metal surface for further oxidation reactions. But synergism provided by Fe and Pt gives better activity in acidic electrolyte as Pt is favourable catalyst for dehydrogenation of methanol in acidic medium. This study will be useful for designing anodic electrocatalysts for MOR.

## Introduction

Over the past few decades, fuel cells (FCs) have become an alternative source of green energy due to the depletion of fossil fuels and an increase in pollution. Among different alcohol-based FCs, extensive study was carried out on DMFC because of its properties such as high energy density (6.1 kWh kg^−1^), low emission of pollutants, low operating temperature, better fuel handling, and processing, etc^1–3^. It has wide applications in the field of electronics, portable devices, automobiles, and transportation^4,5^.

Electrocatalyst is the main component of fuel cells and can also be obstacle in efforts to make fuel cell technology more commercially viable. Pure metal fails to be a strong catalyst for alcohol FCs operation at room temperature because it is easily poisoned by adsorption of carbon monoxide (CO) intermediates. Among the metal catalysts, Pt is considered as the most productive catalyst due to its high activity towards breaking of O–H and C–H bonding present in alcohol molecules. But slow kinetics, high cost and low abundancy of Pt hinders its application in commercial level^6,7^. Also, it faces problems of auto-inhibition or poisoning of catalyst. The CO intermediate is strongly adsorbed and linearly bonded with Pt, which leads to self-poisoning of Pt electrocatalyst. Hence, further adsorption of methanol on poisoned Pt cannot occur, and methanol oxidation drops to a minimum rate^8,9^. So, to reduce this poisoning effect, alloying of Pt with second oxophilic metals such as Fe, Ru, Rh, Mo, Sn, Co etc. are necessary^7,10–13^. The bifunctional effect provided by this alloyed material help in easy adsorption of hydroxyl groups (OH^−^) at more negative potential and catalysed the electrooxidation of adsorbed CO thereby reducing poisoning effect on catalyst. Also, the addition of second metal results in an electronic exchange interaction with Pt and make Pt surface to be less affected by intermediate species^14,15^.

Pt-based bimetallic alloys like PtX (X: Mo, Sn, Co, Ni, Ru, Cu, Fe etc.) show better catalytic activity towards methanol oxidation^10–13^. However, they have their limitations in practical DMFC application because of low durability resulting from dissolution of transition metal during real operating condition and poor chemical stability^16,17^. Among all the bimetallic catalyst, FePt comes as an alternative candidate for methanol oxidation reaction (MOR) because Fe is cheaper than Pt, abundant, good electrical conductor and less toxic in nature^18,19^. They mainly form three structures; one is the disordered face-centred cube also known as A_1_ phase where Fe and Pt atoms are distributed randomly in $\left( {0,0,0} \right), \left( {1/2,1/2,0} \right), \left( {1/2,0, 1/2} \right)$ ${\text{and}} \left( {0, 1/2,1/2} \right)$ crystallographic sites^20–22^. The other two have ordered intermetallic structure such as FePt_3_/Fe_3_Pt and FePt. In ordered FePt structure, i.e., face centred tetragonal (fct) L10 phase, alternating layers of Fe and Pt atoms formed in which Pt occupies sites at $\left( {0,0,0} \right) {\text{and}} \left( {1/2,1/2,0} \right)$, and Fe occupies (1/2, 0, 1/2) ${\text{and}} \left( {0, 1/2,1/2} \right)$ ^23,24^. In A_1_ structure, Fe is difficult to stabilize for electrooxidation reaction, whereas in L10 phase, having high uniaxial magneto-crystalline anisotropy shows better chemical stability^25–27^.

The studies conducted on FePt have shown high catalytic activity over other bimetallic catalysts^18,28^. Also, FePt nanoparticles (NPs) are known to be an excellent CO tolerance catalyst comparable to PtRu^15^. *Lee *et al*.* investigated the performance of binary and ternary catalysts such as FePt, RuPt, MoPt, PtRuFe, and PtRuMo and found that FePt with the content of Fe (28–60%) shows higher catalytic activity than pure Pt^29^. However, Fe loading greater than 60% showed a rapid decrease in catalytic activity and was lower than pure Pt^30^. *Liang *et al*.* also demonstrated comparative analysis of Pt with bimetallic (FePt, NiPt, CoPt) catalyst NPs synthesized by reverse microemulsion route which showed the activity in the order of FePt > CoPt > NiPt > Pt^31^. The result was supported by *Wang *et al*.* through their experimental analysis where they obtained mass activity of FePt (1610 mA/mg of Pt) which is 12 times higher than commercial Pt/C catalyst^32^. Hence, addition of second metals to the traditional catalyst (Pt) can be an efficient strategy to enhance oxidation of methanol. Furthermore, controlling the size, structure and dispersibility of NP in the catalyst preparation process is critical because these parameters are strongly related to the number of active sites on the surface of catalysts. Recently, many research groups have succeeded in improving these properties of catalysts. For example, alloy of Pd e.g.*,* PdM (M = Ni, Co, Cu) and composite with reduced graphene oxide catalyst (rGO) exhibited high electrochemical activity and stability^33^. The transition metal improved the dispersibility of PdM NPs on rGO surface by changing the shape, size, and electronic structure of Pd NPs^34^.

Considering the excellent performance of Fe incorporated with Pt compared to other transition metals from literatures, this research work proposed a facile synthesis route by adopting Fe alloyed with Pt under the presence of PEG-glycerol. Polyethylene glycol (PEG) acts as a protective agent and stabilizes the NPs to prevent agglomeration of FePt NPs during synthesis process. It helps in the reduction of particle size thereby producing uniform NPs with high dispersibility. The electrocatalytic properties of Fe-Pt catalyst was investigated in details for MOR in both acidic and alkaline environment.

## Results and discussion

### Microstructural study

**Figure 1:**
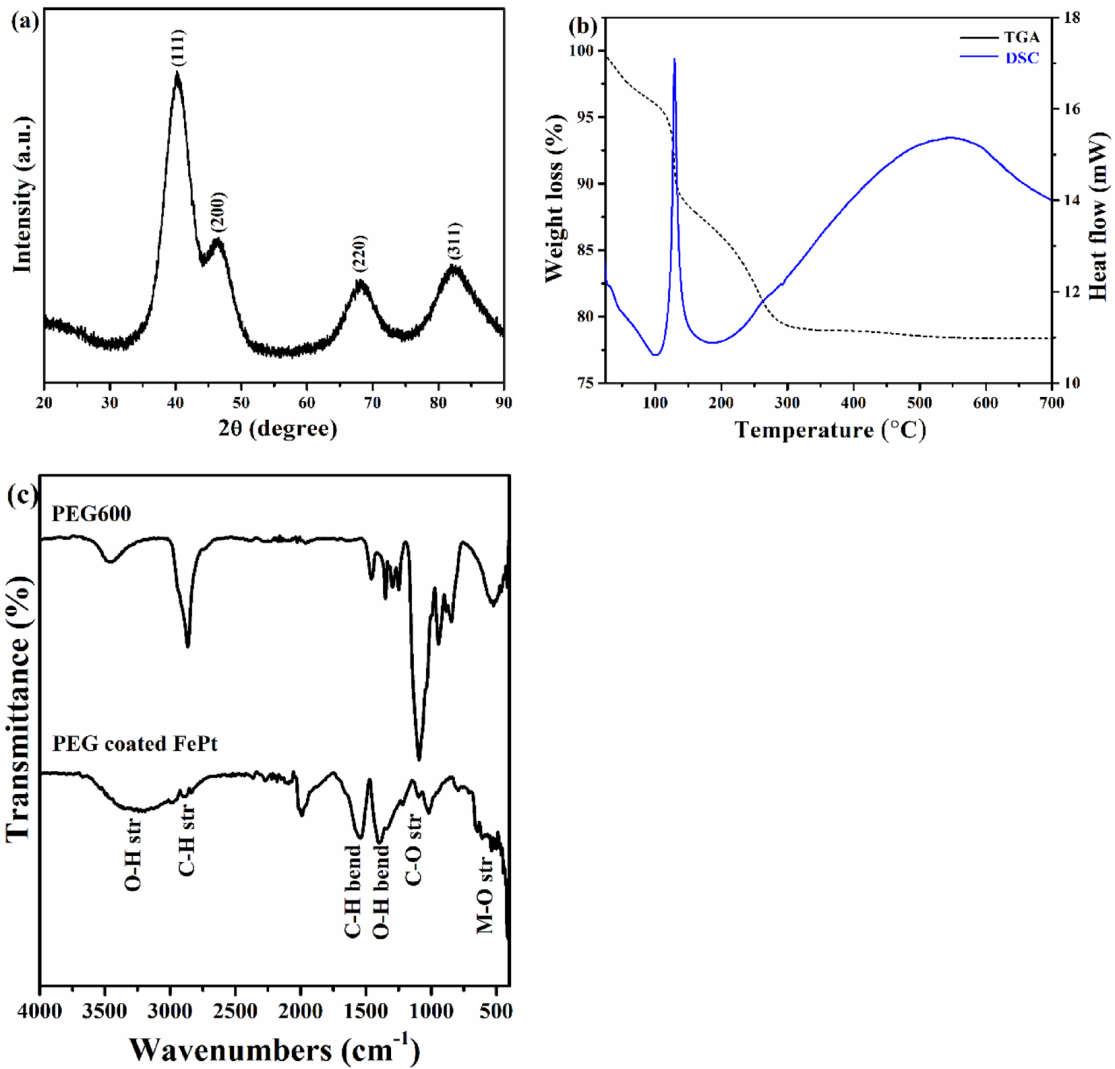
**(a)** XRD pattern, **(b)** TGA/DSC plot and **(c)** FTIR spectrum of FePt NPs. FTIR pattern of PEG-600 is included in fig (**c**) for comparision.

The diffraction peaks in the XRD pattern of the synthesized FePt NPs shown in Fig. 1a are observed at 2θ values of 40.12°, 46.92°, 68.32°, and 82.70° which are correspond to (111), (200), (220) and (311) planes of FePt crystal. These peaks are characteristics of fcc FePt and matches with JCPDS card no. 29-0717. Absence of any additional diffraction peak indicates the sample is pristine and free from contamination. The X-ray diffraction peaks broaden when crystallite become smaller than about a micrometer or when lattice defects are present in abundance^35^. As the nanoparticles are of very tiny around 2.2 nm (according to TEM micrograph) and Fe-Pt alloy is a perfect solid solution, it is expected that the broadening in XRD peak is predominantly due to smaller crystallite size. The average crystallite size (t) was estimated using the Debye–Scherer equation as stated below:

$$t = \frac{K\lambda }{{\beta Cos\theta }}$$

where λ is the wavelength of X-ray (0.154 nm), β represents FWHM and *K* is the Scherrer constant. The calculated average crystallite size was found to be ~ 2.13 nm. The lattice parameter of the catalyst was estimated using following equation:

$$a_{fcc} = \frac{{\sqrt {h^{2} + k^{2} + l^{2} } }}{{2 Sin\theta_{max} }}\lambda$$

where θ_max_ corresponds to the peak position of the highest intensity one. The lattice parameter was found to be 0.388 nm which corresponds to (111) plane of fcc-FePt NPs. The calculated crystallite size from XRD peaks are reported in Table S1.

TGA and DSC profile of the nanomaterial is plotted and shown in Fig. 1b. Nearly 4% weight loss below 120 °C was observed which is attributed to the removal of physically adsorbed water or moisture content. The loss above 120 °C followed by a prominent peak at 129 °C in DSC indicate an exothermic reaction because of the thermal degradation of the polymer. The polymer (PEG) almost burnout till 300 °C and weight loss of about 17% was observed. These results support the surface functionalization of prepared catalyst with PEG molecules.

FTIR analysis was further carried out to investigate the surface functionalization of the polyol on the catalyst surface. Figure 1c shows the FTIR spectra of bare PEG-600 and PEG-coated FePt NPs. A strong and broad band around 3240 cm^−1^ is due to the stretching vibration of O–H group. Doublet bands near to 2887 and 2954 cm^−1^ are correspond to symmetric and asymmetric C–H stretching mode of PEG. Peaks at 1390 and 1525 cm^−1^ are related to the bending vibration of O–H and C–H group respectively. Presence of these peaks confirm the functionalization of FePt NPs with PEG molecules. Also, the characteristic peaks of the PEG polymer appear at around 939 and 1093 cm^−1^. These peaks are corresponding to the C–C and C–O–C stretch, which are suppressed in PEG-coated NPs. The suppression indicates a dipole-cation binding between positive charge of surface metal ions and ether group of PEG molecules^36–39^. The vibrational bands near to 593 and 662 cm^−1^ related to the co-ordination of oxygen with metal^40^. The surface coating of glycols may be explained as the linkage of glycols with change in oxidation state of Pt to Pt^2+^ or Fe to Fe^2+^ at the surface as illustrated in Scheme S1.

**Figure 2:**
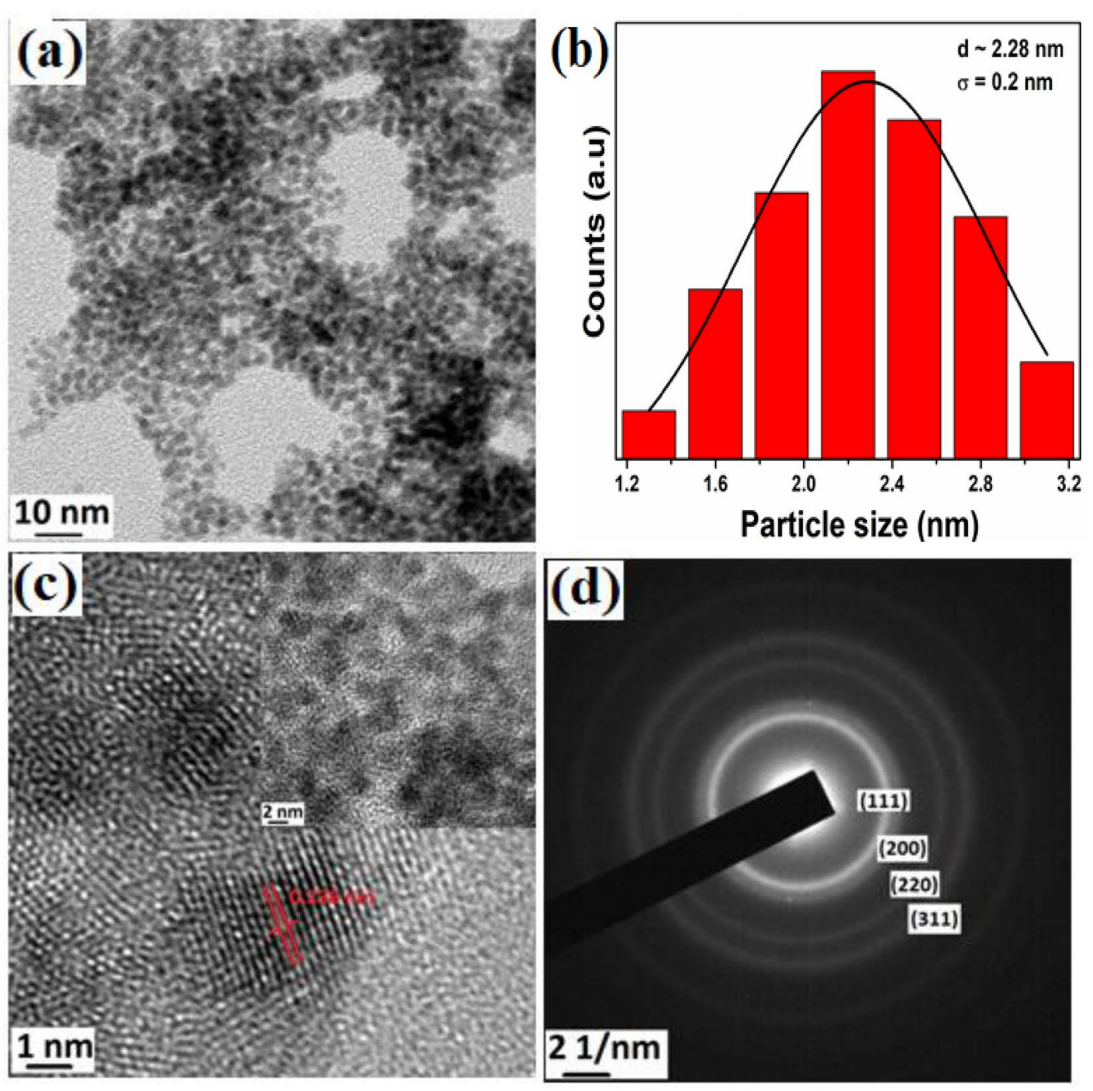
**(a)** TEM image, **(b)** particle size distribution, **(c)** HRTEM image and **(d)** SAED pattern of FePt NPs.

**Figure 3:**
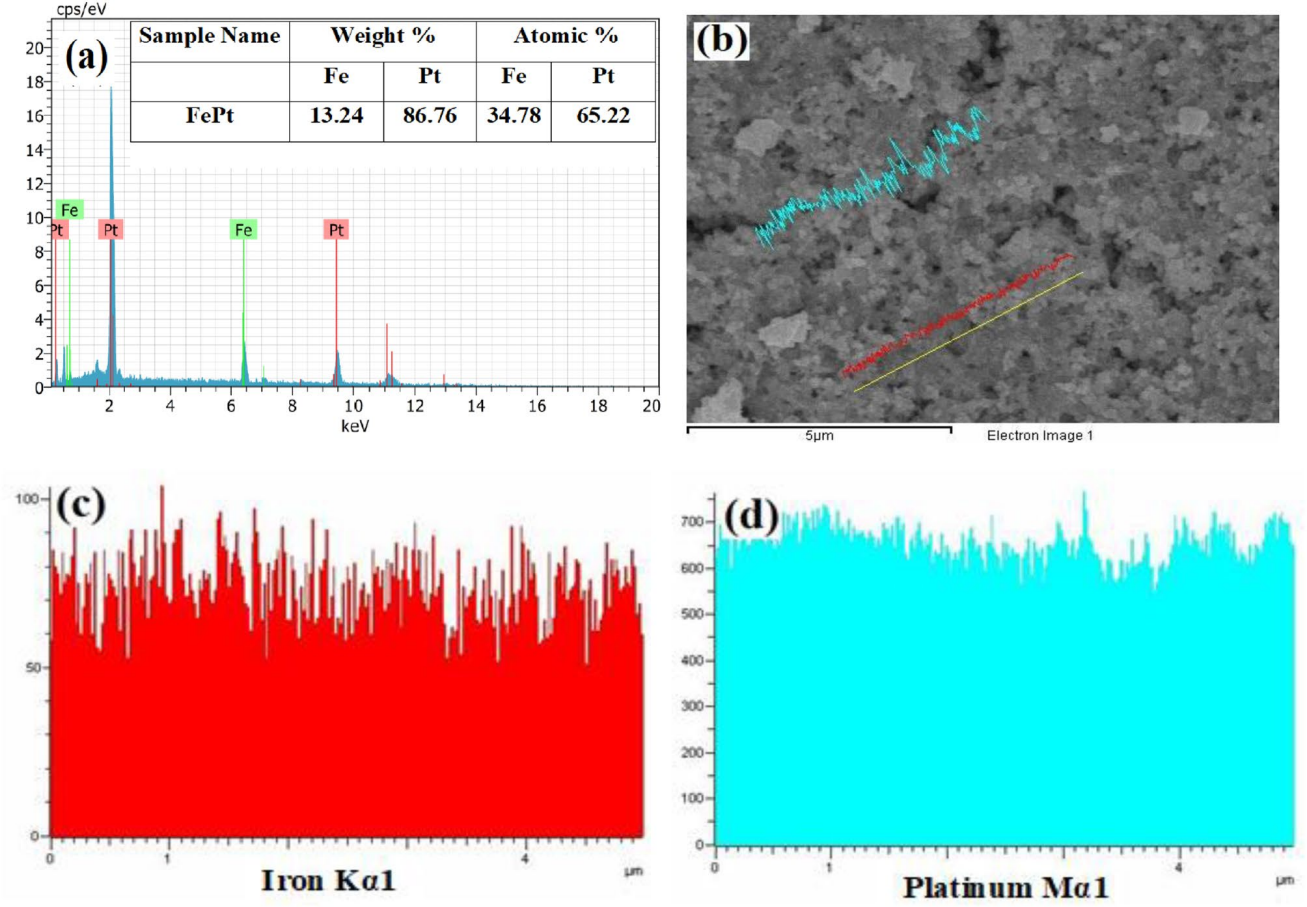
**(a)** EDAX spectrum, **(b)** line scan image and **(c,d)** elemental variation of Fe and Pt in line scan.

FePt NPs exhibits spherical morphology with an average particle size (d) of ~ 2.2 nm with a standard deviation (σ) of 0.2 nm as shown in Fig. 2a. The corresponding size distribution histogram is depicted in Fig. 2b, which indicates that the particles are highly monodispersed. The calculated interplanar spacing of 0.236 nm from HRTEM image (Fig. 2c) is the characteristic of (111) plane and corroborated with the XRD data. SAED pattern shown in Fig. 2d is also matched with XRD data which confirms the formation of fcc-FePt NPs. The EDX profile shows the presence of Fe and Pt (Fig. 3a) which supports the formation of FePt alloy. The line scan profile clearly reveals the homogeneity of Fe and Pt throughout the sample (Fig. 3b–d)_._ The atomic percentage of Pt is higher than the Fe indicating that platinum-rich alloy is formed (Fig. 3a).

**Figure 4:**
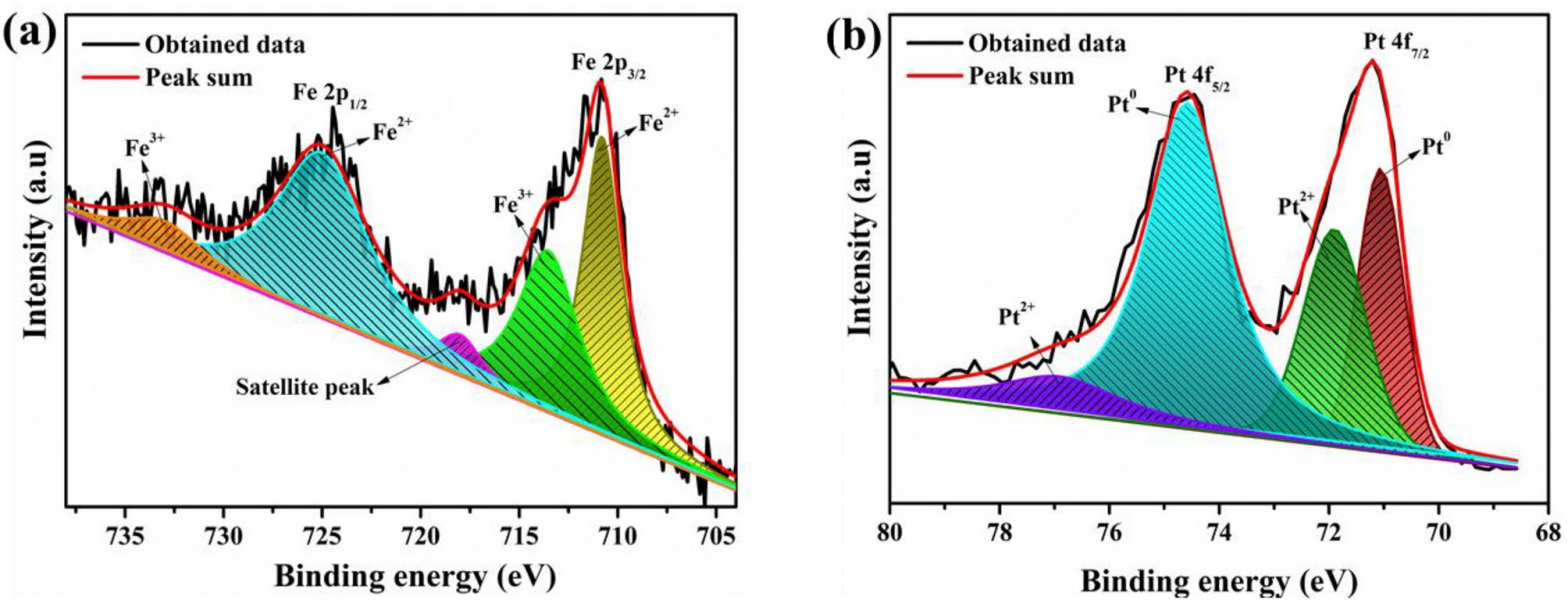
High-resolution XPS spectra **(a)** Fe 2p, and **(b)** Pt 4f of Fe-Pt nanocatalyst.

The XPS analysis was further carried out to examine the chemical composition and oxidation state of Fe and Pt atoms in FePt electrocatalyst. The measurements were performed with a focus on the peaks associated mainly with Fe 2p and Pt 4f, and the core level spectra were calibrated with binding energy (BE) of C 1s (284.6 eV). The high-resolution spectra of Fe 2p and Pt 4f was shown in Fig. 4a,b. Majority of Fe was in oxidized state (Fe^+2^ or Fe^+3^) and Pt was mainly in metallic state. The core spectra of Fe 2p exhibited doublet at BE of 710.9 eV and 725.1 eV, which are assigned to lower energy band (Fe 2p_3/2_) and higher energy band (Fe 2p_1/2_) of Fe^2+^, respectively. The peaks at 713.7 eV and 733.2 eV are related to the Fe^3+^. In addition to this, one satellite peak is also present at 718.2 eV in the case of Fe 2p. The core spectra of Pt 4f exhibited two distinct peaks at BE of 71.06 eV and 74.4 eV which are assigned to Pt 4f_7/2_ and Pt 4f_5/2_, respectively, of pure Pt^0^. In addition to this, two small peaks are also observed at 71.9 eV and 76.9 eV, corresponding to Pt 4f_7/2_ and Pt 4f_5/2_ of Pt^2+^, respectively. Interestingly, the XPS spectra of Pt reveal a slight negative shift as compared to monometallic Pt (71.2 and 74.5 eV respectively) and Fe spectra exhibited a slight positive shift as compared to monometallic Fe (710.6 and 724.6 eV for Fe 2p_3/2_ and Fe 2p_1/2_, respectively)^41,42^. Shifting of position of Fe to higher BE value and Pt to lower BE value indicates the electronic exchange interaction between Fe and Pt^43^. This change in electronic structure shifts the *d*-band centre thereby resulting in changes in adsorption energies of reaction intermediates, which can influence the catalytic activity of the material.

Wettability is a crucial factor for attaining high efficiency and better performance of DMFC at low temperature as hydrophilic anode associated with the lower methanol transfer resistance and facilitate the methanol transport^44,45^. Also, the adsorption and desorption of reactants in catalyst surface under water may change because of the variation of wettability^46^. The hydrophilicity and wettability measurements were done using a contact angle measurement test. It can be observed from the Fig. S1 that the static contact angle reduces from ~ 64° to 30° for the PEG added FePt NPs. A higher contact angle value denotes poor wettability that might lead to reduced access of methanol ions into the pores. Reduction in electrolyte ion accessibility substantially affects surface area utilization which in turn diminishes the performance. Hence, surface modification using PEG is utilized here to increase the hydrophilicity of the FePt NPs. Photographs of water dispersion of PEG coated and bare FePt NPs are also provided in Fig. S2.

### Proposed mechanism of formation of FePt NPs

The formation mechanism of FePt can be explained as follows: it is seen that though Pt(acac)_2_ and Fe(acac)_3_ decompose thermally below 200 °C, Pt-precursor decomposes very fast whereas the later one (iron precursor) takes multiple steps and complete decomposition occurs at relatively high temperature^21^. In the present study, glycerol ((1,2,3-propanetriol) being glycolic compound is used as solvent, reagent, and reducing agent. It is not only used as a solvent but as a co-solvent with water, ethanol, and any other polar solvent, for the synthesis of nanomaterials^47,48^. The hydrogen bonding, polarity, and viscosity play significant role in controlling the size of the NPs. On the other hand, due to Van der Waals attractions between the particles, the dispersion of NPs becomes unstable in glycerol medium and tend to aggregation and coagulation. To minimize this, a long chain polymer PEG600 as a protecting agent is utilized during synthesis stage. When PEG is attached to charge particle surface, the MNPs get stabilized by both steric and electrostatic stabilization thereby prevent agglomeration of the NPs formed. Moreover, it helps in the reduction of the metal precursor by hydroxylation process as per the following reaction:

$${2 }\left[ {{\text{H}} - \left( {{\text{O}} - {\text{CH}}_{{2}} - {\text{CH}}_{{2}} } \right)_{{\text{n}}} - {\text{OH}}} \right] \to {2}\left[ {{\text{CH}}_{{3}} {\text{CHO}}} \right]_{{\text{n}}} + {\text{ H}}_{{2}} {\text{O}}$$

$${\text{M}}\left( {{\text{C}}_{{5}} {\text{H}}_{{7}} {\text{O}}_{{2}} } \right)_{{\text{x}}}\:\xrightarrow[\text {Application of heat energy}]{\text{Presence of Polyol}} {\text{M}}\left( {{\text{OH}}} \right)_{{2}}$$

$${2}\left[ {{\text{CH}}_{{3}} {\text{CHO}}} \right]_{{\text{n}}} + {\text{ M}}\left( {{\text{OH}}} \right)_{{2}} \to {\text{CH}}_{{3}} - {\text{CO}} - {\text{CO}} - {\text{CH}}_{{3}} + {\text{ 2H}}_{{2}} {\text{O }} + {\text{M}}$$

where M denotes the metal (Fe and Pt), and x corresponds to the number of moles of acetylacetonate group attached to the metals. Finally, Fe and Pt diffuse to form an alloy under an inert atmosphere. It is also reported that Pt nuclei form in the early stage of reaction helps in catalytic decomposition of Fe precursor^49^. So, it can be inferred that Pt(acac)_2_ reduces easily in polyol solvent under the application of heat whereas Fe(acac)_3_ follows complex reduction process. Hence, Pt-rich FePt was formed in this method in spite of using 2:1 molar ratio of Fe to Pt precursor which is also supported by the EDX result.

### Electrochemical analysis

#### Electrocatalytic oxidation of methanol

**Figure 5:**
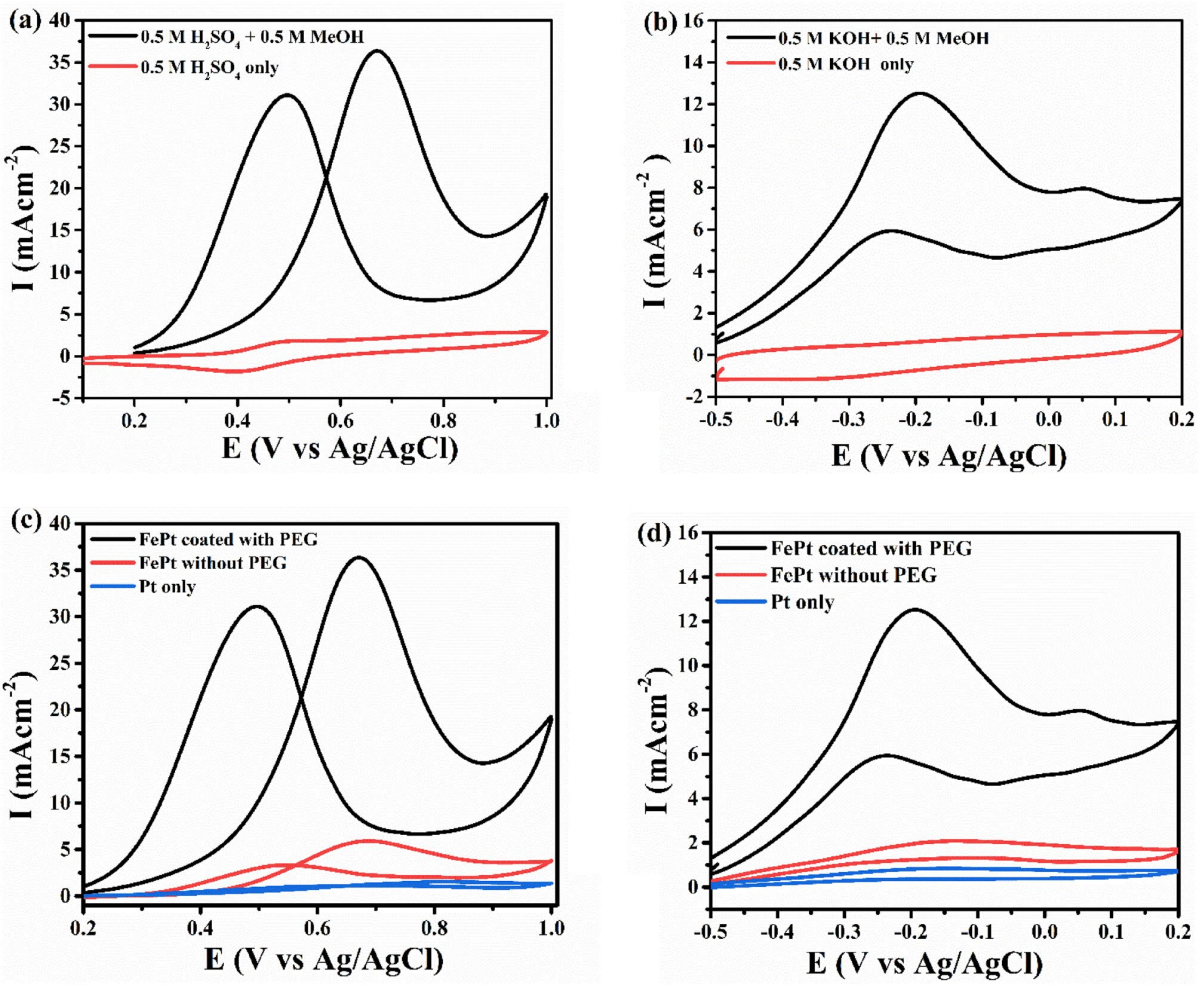
CV plots of functionalized FePt NPs in **(a)** 0.5 M H_2_SO_4_ and **(b)** 0.5 M KOH with and without methanol. The comparison plots of functionalised FePt, bare FePt and Pt is shown in **(c)** acidic, and **(d)** alkaline medium at scan rate of 50 mV s^−1^. Current is normalized by geometric surface area used which is 0.07 cm^2^.

Initially, the electrocatalytic activity of the catalyst was investigated with or without methanol at room temperature for both the media. The current axis is normalized by the geometric surface area of electrode used *i.e.* 0.07 cm^2^. The addition of methanol results in a pair of redox peaks as observed in Fig. 5a,b. For comparative analysis, we considered bare Pt and FePt without PEG along with FePt coated with PEG (Fig. 5c,d) and it is observed from the CV curve that the catalyst coated with PEG 600 shows higher oxidation peak as compared to other two which follows the order of FePt_coated with PEG_ > FePt_without PEG_ > bare Pt. It can be explained as the PEG acts as protective agent and stabilizes the NPs to prevent agglomeration during synthesis process. It helps in the reduction of particle size thereby producing uniform NPs. Due to the high specific surface area of the tiny NPs, when exposed to reactant (in this case methanol) trigger the reaction and enhances the oxidation process. Further, the utilization of both PEG and glycerol soluble alcohols increases the hydrophilicity of FePt catalyst validated by the contact angle measurement which enhances the performance of the catalyst. In addition, onset potential is considered as an important parameter on which activity of the catalyst lies. Low onset potential with high current density is a characteristic property of a good catalyst. As observed from Fig. 5, the oxidation of methanol occurs at the onset potential of 0.33 V and −0.46 V in acidic and alkaline media respectively, which signify FePt NPs is a better catalyst than pure Pt which has the onset potential of ~ 0.45 V and − 0.48 V (from Fig. 5c,d) in respective medium. Better catalytic activity of alloy as compare to the pure metal is mainly due to the bifunctional effect and electronic exchange interaction between Fe and Pt as supported with the XPS data. Here, a significant oxidation peak is observed in an anodic sweep at ~ 0.671 V for acidic and ~  − 0.193 V at 50 mV s^−1^ for alkaline media in the presence of methanol and the peak current corresponding to that is represented by I_f_. In the backward sweep, there is a peak at ~ 0.49 V and − 0.24 V in the acidic and alkaline electrolyte, which arises due to the removal of intermediate carbonaceous species. The corresponding peak current is called backward current and is denoted by I_b_. From Fig. 5, acidic environment is found to be superior for FePt catalyst in terms of higher anodic oxidation current. The higher current response refers that large area of the catalyst are accessible to electrolyte. Fig. S3 shows that the PEG-Glycerol mediated FePt bimetallic catalyst exhibited excellent performance in both acid and alkaline media as compared to bare Pt and FePt NPs.

The electrochemical reaction mechanism on the surface of catalyst can be explained as follows: in the forward sweep, the chemisorption followed by decomposition of methanol results in by-products such as CO and intermediate species which has strong affinity towards Pt^24^. This reduces the life span of catalyst resulting in lower efficiency. During the reverse scan, the “poisoning” species get oxidized in the presence of adsorbed OH^−^ results in regeneration of active sites. In bimetallic NPs such as FePt, Fe acts as a promoter for dissociating the water to hydroxyl ions which helps in conversion of CO to CO_2_ over catalyst surface. This enhances the performance and provides stability to platinum catalyst.

#### Effect of scan rate

**Figure 6:**
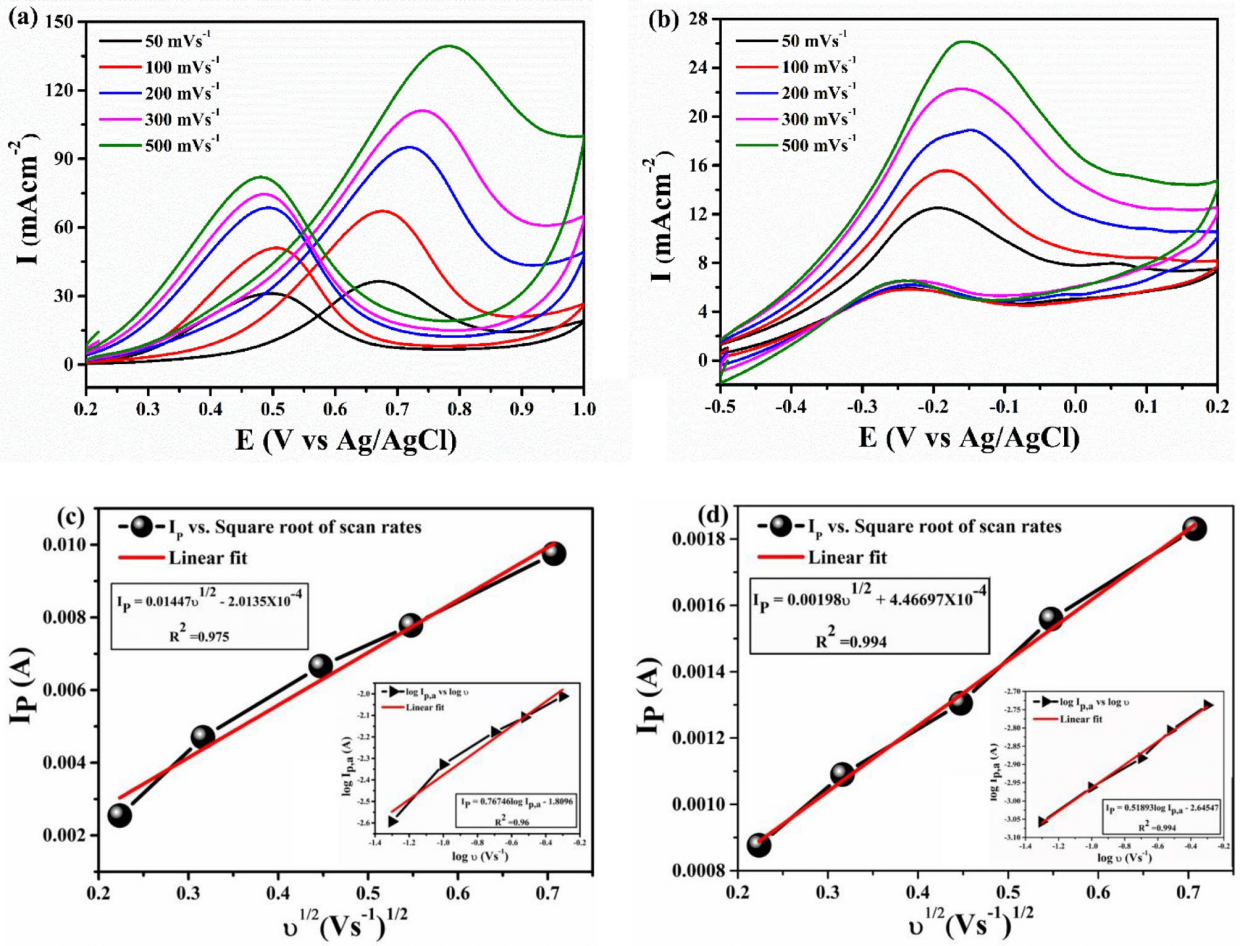
CV curves at different scan rates in **(a)** acidic medium, **(b)** basic medium and **(c,d)** anodic peak current versus square root of scan rates in respective media (log I_f_ versus log ν have been plotted in the insets of **(c,d)**).

The effect of scan rate on voltammetric behaviour of FePt in acidic and alkaline media was investigated using cyclic voltammetry (Fig. 6a,b). At different scan rate, both forward and backward current increases linearly suggesting a behaviour consistent with surface confined voltammetry. To further examine the electrochemical behaviour of FePt, the influence of scan rates on peak potential was analysed. With increase in scan rate, anodic peak potential shifted towards positive potential and a linear relationship was obtained in range of 10–500 mV s^−1^ (Fig. 6c,d) which implies that the oxidation process is controlled by diffusion of methanol in both media^49^. Moreover, the plots of log I_f_ versus log ν also showed a linear relationship at different scan-rate with a slope of 0.76 (R^2^ = 0.96, inset of Fig. 6c) and 0.51 (R^2^ = 0.994, inset of Fig. 6d) for acidic and alkaline medium respectively. Theoretically, the slope lies in between 0.5 and 1.0 is considered as value for diffusion and adsorption-controlled mechanism^50,51^. Here, the obtained slope values indicate the contribution of both diffusion and adsorption phenomenon to oxidation mechanism. Moreover, the presence of only oxidation peak in both anodic and cathodic sweeps suggest the methanol oxidation on FePt surface is an irreversible process and this can be validated from the linear relationship between I_f_ and ln ν as shown in Fig. S4.

Figure S5a,b depicts the variation of I_f_ and I_b_ concerning scan rate in acidic medium and alkaline medium respectively. It is clear from the graph that as the scan rate increases both I_f_ and I_b_ increases but the rate of I_b_ does not increase significantly. As back current arises due to the removal of carbonaceous species, therefore, lower back current is a good agreement to catalyst’s poison tolerance capacity. From Fig. S5b, we observed that I_f_ increases linearly with scan rate in alkaline medium, but the current is low as compared to acidic medium. This implies that catalyst is less active in alkaline medium. The tolerance ratio was calculated by the ratio of I_f_/I_b_. Out of two, alkaline medium showed slightly higher tolerance ratio of 1.91 than acidic medium which is 1.16. High I_f_/I_b_ in alkaline medium indicates the better removal of carbonaceous species during MOR due to additional adsorption of OH^−^ ions provided by KOH from electrolyte solution. The lower tolerance ratio in the acidic medium can be related to the adsorption of bi-sulphate ions on the active sites of the nanocatalyst, which hinders further oxidation of intermediates. Interestingly, forward peak current which corresponds to the methanol electro-oxidation is 12.52 mA cm^−2^ for alkaline electrolyte which is approximately three times lower than that in acidic electrolyte (36.35 mA cm^−2^ at 50 mV s^−1^), thereby making alkaline medium less favourable for methanol oxidation using FePt electrocatalyst. Furthermore, the mass activity is also found to be higher in acidic medium i.e. 80.09 mA mg^−1^ than alkaline one (28.28 mA mg^−1^). These results support that Fe has high compatibility in oxidizing methanol when paired with Pt in low pH environment. Composition of electrode and potentio-dynamic analysis obtained from CV are presented in Table 1.

**Table 1:** Composition of electrode and potentiodynamic analysis of FePt catalyst towards methanol oxidation.

Electrodes	At% of Pt	At% of Fe	Operating condition		Potentiodynamic analysis (at 50 mV s^−1^)						
			CH_3_OH conc	Supporting electrolyte	Mass activity (mA mg_Pt_^−1)^	E_onset_ (V)	j_f_ (mA cm^−2^)	j_b_ (mA cm^−2^)	E_f_ (V)	E_b_ (V)	j_f_/j_b_
FePt/GCE	65.22	34.78	0.5 M	0.5 M H_2_SO_4_	80.09	0.33	36.35	31.08	0.67	0.49	1.16
FePt/GCE	65.22	34.78	0.5 M	0.5 M KOH	28.28	− 0.46	12.52	6.53	− 0.19	− 0.24	1.91

#### Chronoamperometry analysis

**Figure 7:**
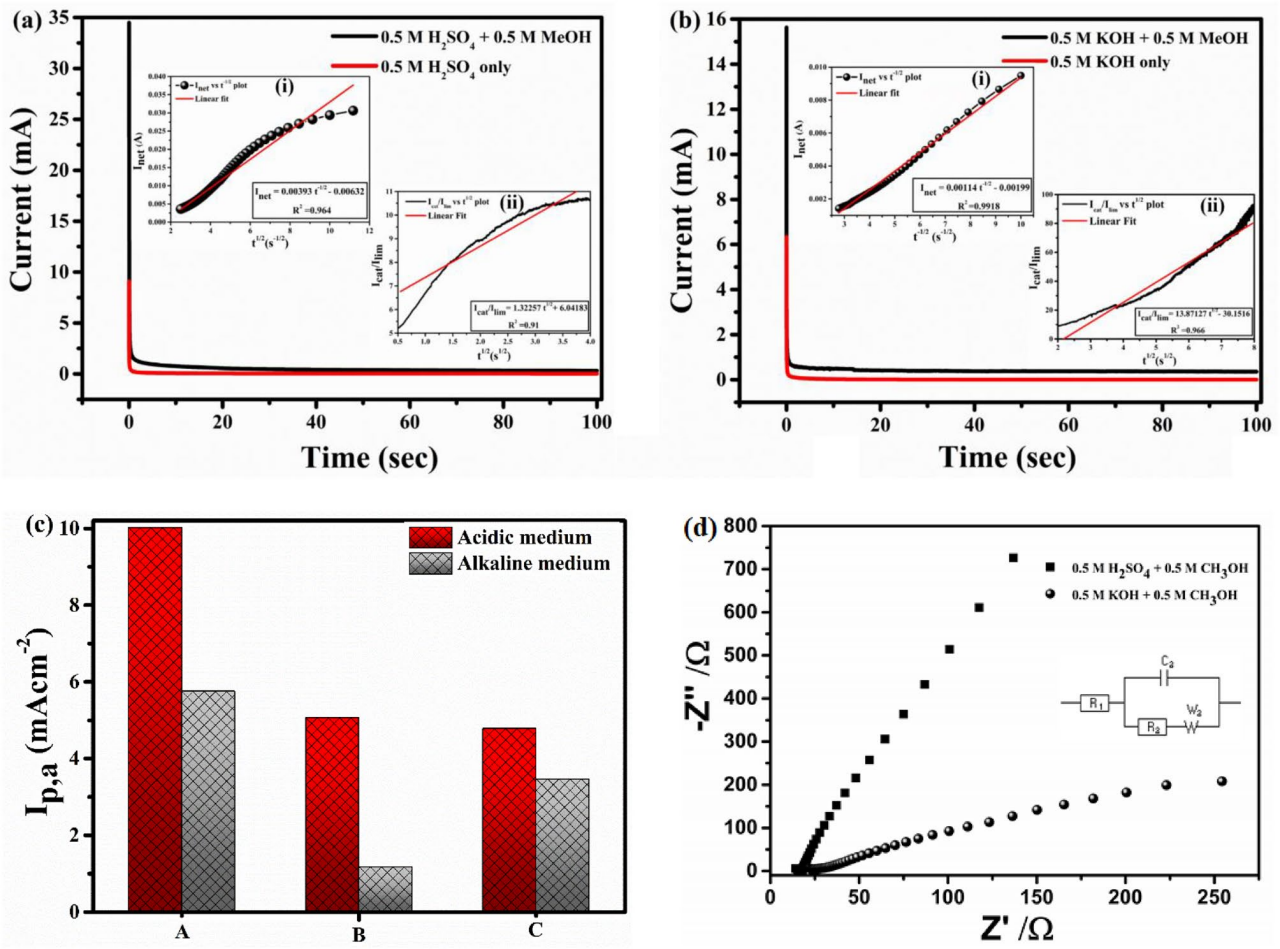
Chronoamperograms of FePt NPs in **(a)** 0.5 M H_2_SO_4_, **(b)** 0.5 M KOH with and without methanol. Insets: **(i)** plot of I_net_ Vs t^−1/2^ and **(ii)** I_cat_/I_L_ Vs t^−1/2^ for respective media, **(c)** stability and reusability analysis of catalyst for 300 cycles. *Column A:* the initial cycle, *Column B:* the 300th cycle and *column C:* the 301th cycle in freshly prepared electrolyte of both the medium at scan rate of 50 mV s^−1^ and (**d**) Nyquist plot of FePt NPs in 0.5 M H_2_SO_4_ / 0.5 M CH_3_OH and 0.5 M KOH / 0.5 M CH_3_OH and its equivalent circuit diagram.

Chronoamperometry was employed to gather additional information about the electro-oxidation of methanol. Figure 7a,b shows the i-t curves of the catalyst for 100 s in both the media in presence and absence of 0.5 M CH_3_OH. The catalytic rate constant (k_cat_) and diffusion co-efficient of methanol were estimated from chronoamperometry profile. The slope in the linear portion of I_net_ versus t^−1/2^ (insets of Fig. 7a,b) is used to estimate the diffusion coefficient (D) in conjugation with Cottrell equation:

$$I_{net} = nFAD^{1/2 } c\pi^{ - 1/2} t^{ - 1/2}$$

where F, n, A, t, c is Faraday's constant, number of electron transfer during methanol oxidation, area of the working electrode (0.07 cm^2^), time and methanol concentration respectively. The mean diffusion coefficient of methanol is calculated to be $4.26 \times 10^{ - 6} { }$ cm^2^ s^−1^ for acidic medium and $3.58 \times 10^{ - 7} { }$ cm^2^ s^−1^ for alkaline medium. The diffusivity of methanol in acidic medium is better than the basic one. Higher diffusivity implies higher oxidation current and displayed comparable results obtained from CV analysis in Fig. 6. The rate constant was calculated using this equation:

$$I_{Cat} /I_{L} = \pi^{{{\raise0.7ex\hbox{$1$} \!\mathord{\left/ {\vphantom {1 2}}\right.\kern-0pt} \!\lower0.7ex\hbox{$2$}}}} (k_{Cat} ct)^{{{\raise0.7ex\hbox{$1$} \!\mathord{\left/ {\vphantom {1 2}}\right.\kern-0pt} \!\lower0.7ex\hbox{$2$}}}}$$

where I_cat_ and I_L_ represent the current in presence and absence of methanol respectively. I_cat_/I_L_ versus t^1/2^ is plotted in the inset (ii) of Fig. 7a,b. The value of k_cat_ is calculated to be $1.13 \times 10^{3} \;{\text{cm}}^{3} \;{\text{mol}}\;{\text{s}}^{ - 1}$ for acidic and $3.49 \times 10^{2} \;{\text{cm}}^{3} \;{\text{mol}}\;{\text{s}}^{ - 1}$ for alkaline media. Higher catalytic rate constant in case of acidic medium reveals faster conversion of methanol molecules on catalyst surface to product per active site per unit time, thereby making FePt suitable for developing electrocatalyst in PEM- based DMFC. The obtained data are presented in Table 2 for both the media.

**Table 2:** Data obtained from chronoamperometry study (at 50 mV s^−1^).

Electrodes	Medium	Chronoamperometry current density (mA cm^−2^)	Diffusion co-efficient (cm^2^ s^−1^)	Rate constant (cm^2^ mol^−1^ s^−1^)
FePt/GCE	Acidic	5.11	4.26 × 10^–6^	1.13 × 10^3^
FePt/GCE	Alkaline	4.58	3.58 × 10^–7^	3.49 × 10^2^

In addition to this, long term cycle stability and reusability of catalyst is very important for practical application. In this work, stability test of FePt catalyst has been carried out using cyclic voltammetry in 0.5 M H_2_SO_4_ + 0.5 M CH_3_OH and 0.5 M KOH + 0.5 M CH_3_OH solutions for consecutive 300 cycles and corresponding plot is depicted in Fig. 7c. It was observed from the plot that peak anodic current obtained from forward sweep of CV curve gradually reduces with increasing cycle number. Compared to initial cycle, the activity of catalyst is reduced by 49.4% for acidic and 79.5% for alkaline electrolyte at 300th cycle. This reduction may cause due to the accumulation of toxic species (carbonaceous intermediates) on the catalyst surface or excess consumption of methanol during consecutive scans. So, to examine the effect of methanol consumption, the catalyst was reused again in freshly prepared electrolyte in both the medium. The peak current density for new electrolytes after 300th cycle obtained to be 4.78 mA cm^−2^ (nearly 52.25% of initial cycle) and 3.47 mA cm^−2^ (nearly 39.72% of initial cycle) for acidic and alkaline electrolyte respectively. This indicates that methanol consumption maybe the primary reason for decreasing current density in the ongoing 300 cycles. After continuous 300 cycles test, the structure, morphology and chemical stability of the catalysts was investigated using TEM and XRD. The TEM imaging indicates no significant morphological changes on the surface of the catalyst after 300 cycles stability test (Fig. S6b). The catalyst retained its original spherical structure even after 300 cycles in alkaline medium with an average particle size of ~ 2.2 nm which was comparable to that of fresh catalyst (Fig. S6a). The chemical stability of the FePt catalyst coated with PEG was examined by the XRD on samples tested to acidic (0.5 M H_2_SO_4_ solution, pH = 2.4) and alkaline (0.5 M KOH solution, pH = 13.7) electrolytes for 300 consecutive cycles. XRD (Fig. S6c) studies revealed no significant changes in the diffraction patterns. The sample retained its original structure in KOH solution (pH = 13.7) but crystallinity is marginally decreased in the acidic solution. The above results reveal the good chemical stability of FePt material towards methanol oxidation. Moreover, in terms of electrochemical stability, the material exhibited only 20.5% decay after 300 potential cycles in an alkaline medium than PANI-NRGO/CoPd which resulted in about 21% decay after 155 potential cycles in 0.5 M KOH and 1 M MeOH solution^52^. So, the excellent long-term stability of this catalyst proved to be a useful alternative for DMFC application.

#### Electrochemical impedance study (EIS)

EIS analysis was carried out to study the charge transfer kinetics and the electrochemical behaviour of the synthesized nanocatalyst. The Nyquist plot and equivalent fitted circuit are given in Fig. 7d. R_1_ denotes the series resistance, which may arise due to (i) electrolyte resistance, (ii) contact resistance between electrode and electrolyte interface, and (iii) intrinsic electrical resistance of the material. R_1_ value is lower in the acidic electrolyte (H_2_SO_4_) compared to its alkaline (KOH) counterpart. The lower value denotes better interaction in electrode/electrolyte interface, which enhances the performance in the acidic medium. R_2_ is the charge transfer resistance, which indicates the chemical kinetics of the corresponding system. Lower R_2_ value highlights faster charge transport behavior in the acidic medium. More rapid reaction kinetics is essential for the better catalytic activity, and this proves that the FePt system performs better in the H_2_SO_4_ as compared to KOH environment. Another important parameter obtained from EIS is the double layer capacitance denoted as C_2_. Double layer capacitance is depending on the ion accumulation at the electrode/electrolyte surface. The lower value of C_2_ signifies the dearth of accumulated ions on the FePt/KOH surface, which can relate with the absence of semi-circle in the Nyquist plot. The analysed data are summarised in Table 3.

**Table 3:** Parameters derived from the EIS data using EC Lab software.

Parameters	Units	H_2_SO_4 _	KOH
R_1_	Ohm	9.80	15.19
R_2_	Ohm	0.03	6.46
C_2_	F	0.22 × 10^–3^	76.45 × 10^–9^

#### Effect of methanol concentration

**Figure 8:**
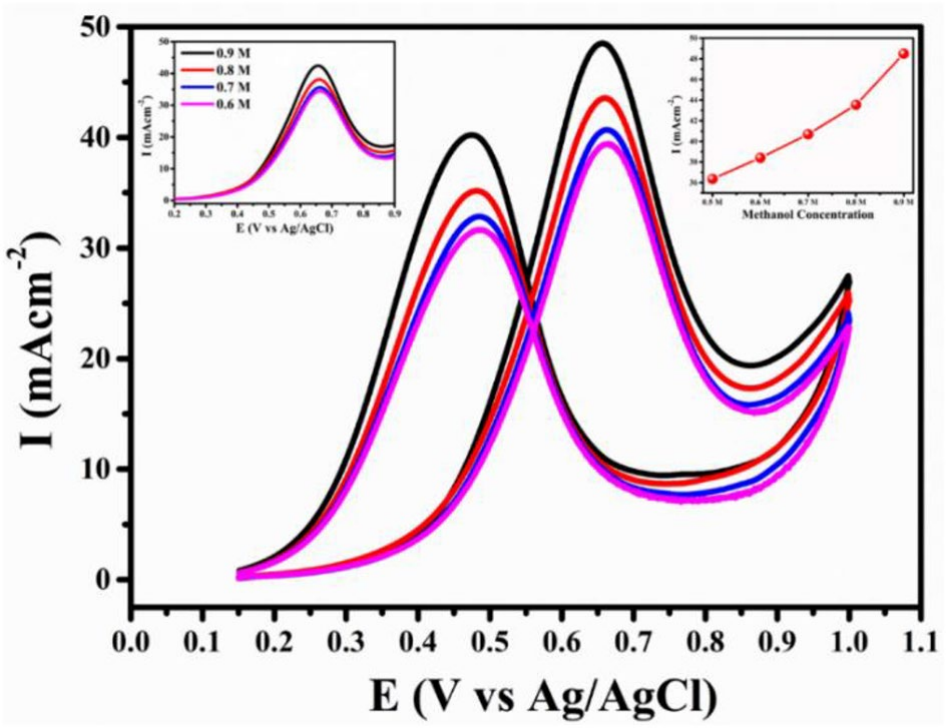
CV response for various concentration of methanol at a scan rate of 50 mV s^−1^ in acidic medium. Inset **(i)** LSV and inset **(ii)** anodic current versus methanol concentration.

The significance of methanol concentration on the electrocatalytic activity of FePt NP was carried out in acidic environment by subsequently adding methanol. The I_f_ and I_b_ peaks increase with increasing methanol concentration up to 0.9 M as shown in Fig. 8. After that the peak current stops increasing significantly, this effect is assumed to be due to the saturation of active sites of the catalyst surface. Broadening of the peak was also observed in the plot which may be due to the accumulation of unoxidized methanol molecules on the catalyst surface.

## Conclusion

In this work, we have demonstrated a facile method to synthesize monodispersed and a highly dispersed fcc-FePt catalyst using PEG-Glycerol route. FePt possesses efficient electrocatalytic behavior towards methanol oxidation in acidic medium. Three-fold increment in current density was observed in case of acidic as compared to alkaline one. Improved current density in low pH is attributed to the better diffusivity and high catalytic rate constant $\left( {1.13 \times 10^{3} \;{\text{cm}}^{3} \;{\text{mol}}\;{\text{s}}^{ - 1} } \right)$ of catalyst thereby making it more favourable for acidic medium. In addition, the second metal (Fe) optimizes the crystal structure and electronic properties of Pt, which mostly exists as Pt-M alloy. The synergistic impact of Fe and Pt helps in the oxidation of carbon containing intermediate species adsorbed on catalyst surface, helps in regenerating more active sites for subsequent reactions. Therefore, this work validates that synthesis of FePt catalyst in presence of PEG as protective agent can be considered as suitable technique to prepare uniform hydrophilic catalyst for methanol oxidation reaction.

## Materials and methods

### Chemicals and reagents

Platinum (II) acetylacetonate (Pt(acac)_2_, 97%), iron (III) acetylacetonate (Fe(acac)_3_, 97%), sulphuric acid (H_2_SO_4,_ 98%) and nafion solution (5 wt. % perfluorinated) were purchased from Sigma Aldrich. Polyethylene glycol (PEG, 98%), potassium hydroxide (KOH, 85%), methanol (CH_3_OH, 99%), ethanol (C_2_H_5_OH, 99%) and glycerol (C_3_H_8_O_3_, 99%) were procured from SD Fine Chemical Limited. DI water was used in synthesis and testing. All the chemicals were used without any further processing.

### Preparation of FePt nanoparticles

FePt NPs were prepared using glycerol as solvent and glycol (PEG-600) as reducing agent as well as surfactant. Initially, 50 mL of glycerol was heated to a temperature of 100 °C for 30 min with stirring. Then, 0.02 mol of Fe(acac)_3_ and 0.01 mol of Pt(acac)_2_ were dissolved in the above solvent. After that 10 mL of PEG-600 was added drop wise to the solution. The entire procedure was carried out in nitrogen atmosphere. The solution was then refluxed for 3.5 h at 220 °C. Later, the sample was collected via centrifugation and washed several times with DI water and ethanol mixture. Finally, the precipitate was oven dried for characterization and testing. Similar preparation method was adopted to synthesize Pt and bare FePt (without PEG 600) NPs for comparison.

### Material characterizations

Morphological analysis of the prepared material was done by TEM (transmission electron microscope, JEOL JEM-2100F) equipped with HRTEM and SAED (selected area electron diffraction). The crystallinity, phase, and purity of NPs were studied by XRD (D8 Advanced Bruker) with CuKα radiation (λ = 1.54 Å) operating at 40 mA and 30 kV. Debye–Scherrer relation was used for the calculation of crystallite size (t). The presence of functional group was examined by fourier transform infrared spectrum (IR Affinity-1) recorded in KBr disc. Thermal analysis (SDT Q60, TA Instruments, USA) of the synthesized sample was done in a nitrogen atmosphere by heating the sample from room temperature to 700 °C (10 °C/min). The elemental composition of the sample was verified by EDX equipped with SEM (Zeiss, EVO 18) instrument. The elemental oxidation state of FePt catalyst was analysed by X-ray photoelectron spectroscopy (XPS) (Model: PHI5000VERSAPROBE III, Make: ULVAC-PHI, Inc., Japan). Electrocatalytic activity of prepared catalyst towards methanol was done by the electrochemical workstation (CHI 660c).

### Electrochemical analysis

The electrochemical activity of the catalyst was carried out in a three-electrode based electrochemical workstation (CHI 660c, USA). FePt coated glassy carbon electrode (GCE), Ag/AgCl and Pt wire were used as working, reference and counter electrode respectively. The working electrode was prepared by coating 12 µl of NPs suspension (1.5 mg FePt NPs dispersed in 500 µl ethanol). Then, 5 µl of nafion solution was dropped onto the prepared electrode which acts as a binder. The use of nafion is mainly to prevent the leakage of the catalyst during the electrochemical testing. The catalytic activity for methanol oxidation was measured in two different media i.e. 0.5 M H_2_SO_4_ (acidic) and 0.5 M KOH (alkaline) in presence of 0.5 M CH_3_OH at room temperature. Cyclic voltammetry (CV) study was carried out at scan rate ranging from 10 to 500 mVs^−1^ in a potential window of 0.1 to 1.1 V and − 0.5 to 0.2 V for acidic and alkaline medium, respectively. Chronoamperometry (CA) curve was recorded for both the medium to check the stability of catalyst during MOR. Electrochemical impedance spectroscopy (EIS) test was conducted at an excitation voltage of 5 mV in frequency range of 0.1 to 10^5^ Hz. Finally, the effect of methanol concentration on catalytic activity of FePt NPs was examined for acidic medium.

### Supplementary Information

Supplementary Information.

## Data Availability

All data generated/analysed during the present work are included in this manuscript.
