# Supplementary material for: Facile synthesis of PEG-glycerol coated bimetallic FePt nanoparticle as highly efficient electrocatalyst for methanol oxidation
Source: Sci Rep. 2023 Aug 15;13:13249. doi: 10.1038/s41598-023-38358-5 (PMC10427643; doi:10.1038/s41598-023-38358-5)
Supplement: Supplementary file 1 — Supplementary Information. [file 41598_2023_38358_MOESM1_ESM.docx]

**Supplementary Information**


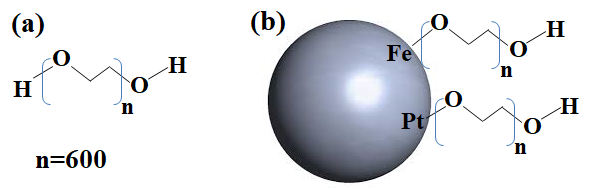


**Scheme S1**: Schematic of binding of glycols on FePt surface.

**Table S1:** Calculated crystallite size from XRD peaks using Debye Scherrer formula.

| **Plane** | **2θ/ degree** | **FWHM**  **(β)** | **Crystallite size/ nm** |
| --- | --- | --- | --- |
| (111) | 40.12 | 3.95 | 2.13 |
| (200) | 46.92 | 3.24 | 2.67 |
| (220) | 68.32 | 5.10 | 1.88 |
| (311) | 82.71 | 6.71 | 1.57 |


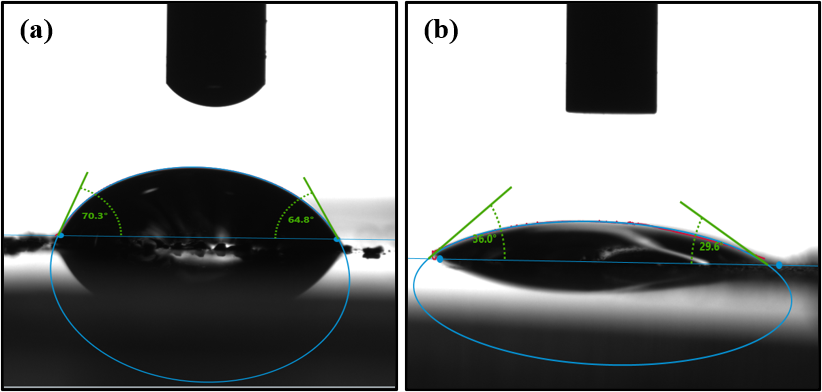


**Figure S1:** Contact angle measurement of FePt NPs prepared in **(a)** absence of PEG, and **(b)** presence of PEG.


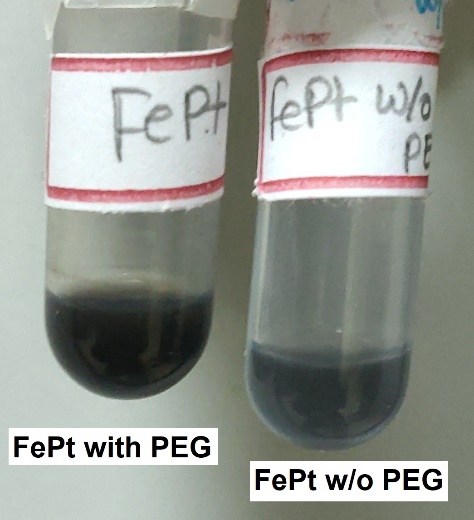


**Figure S2:** Photographs of water dispersion of PEG coated and bare FePt NPs.

**
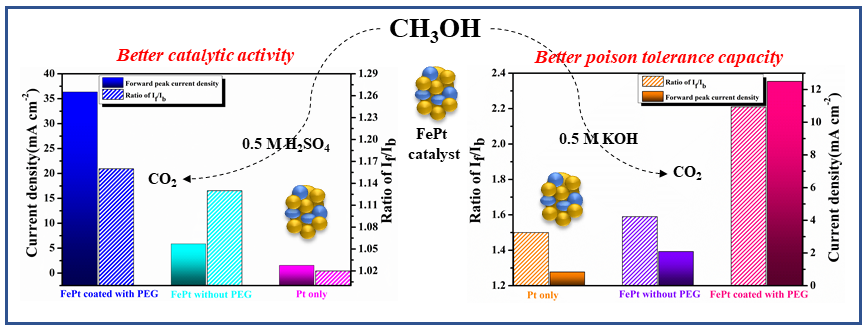
**

**Figure S3**: Comparison of electrocatalytic parameters of prepared NPs in both media.


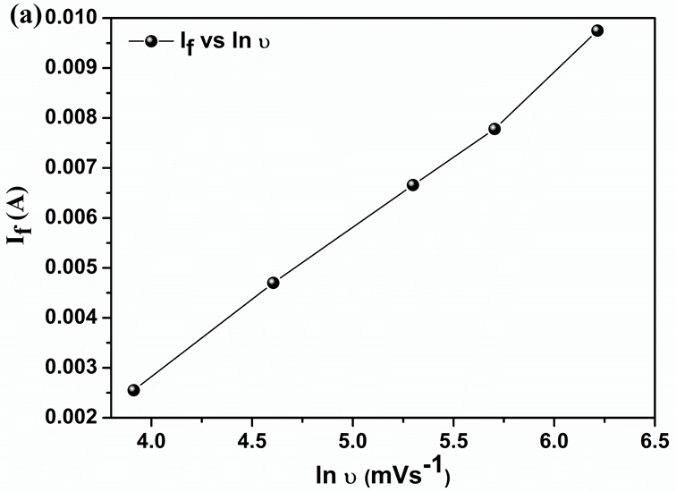

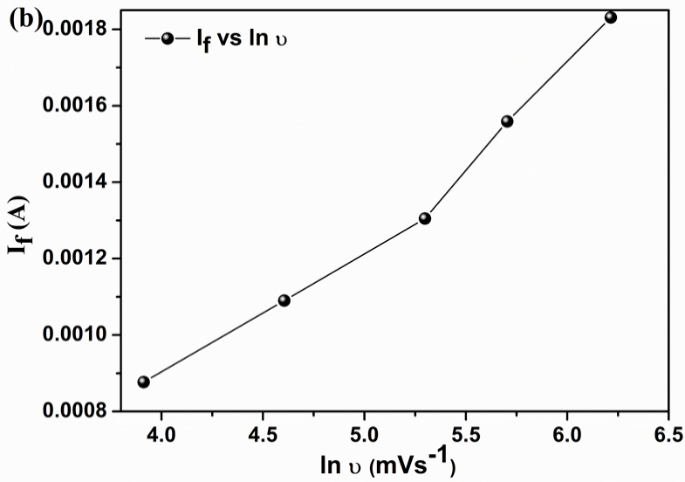


**Figure S4:** Forward peak current vs natural logarithm of scan rate in **(a)** acidic medium and **(b)** alkaline medium.


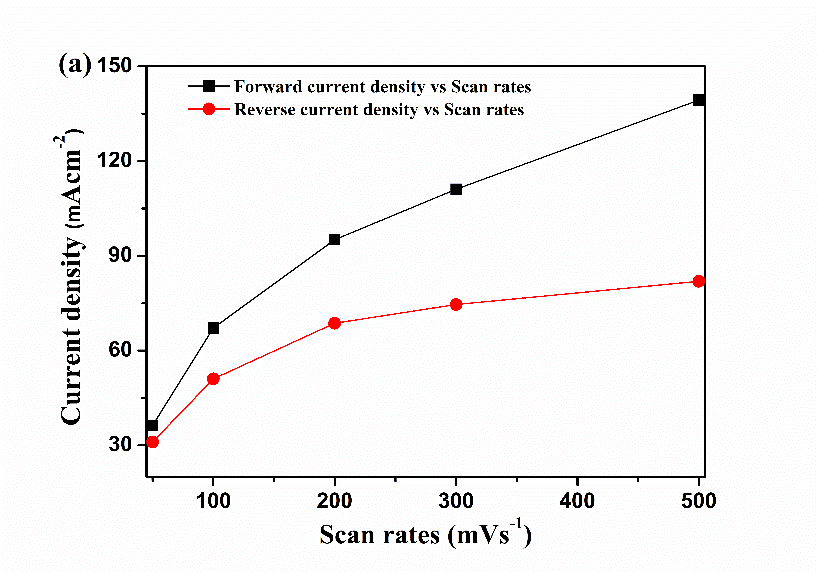

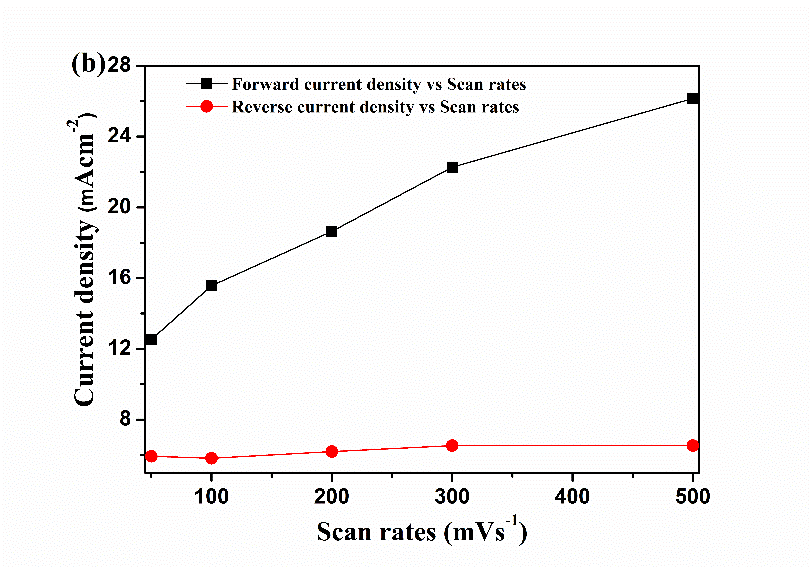


**Figure S5:** CV graphs of forward and reverse current densities vs scan rates in **(a)** acidic medium and **(b)** alkaline medium.


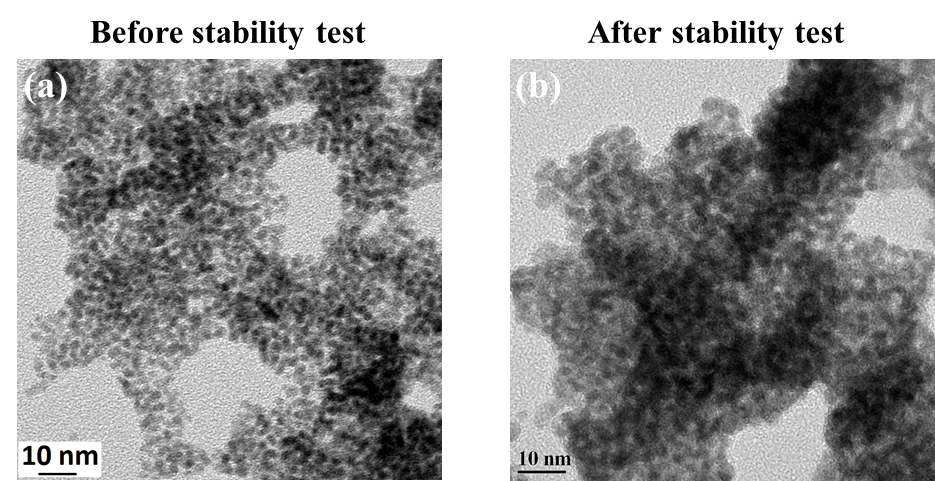


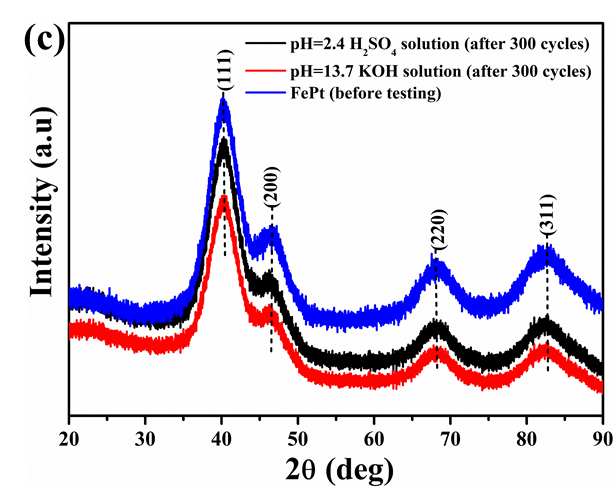


**Figure S6.** TEM images of FePt catalyst **(a)** before and **(b)** after 300 cycles stability test and **(c)** XRD patterns of FePt under different conditions.
